# Up-skilling associate clinicians in Malawi in emergency obstetric, neonatal care and clinical leadership: the ETATMBA cluster randomised controlled trial

**DOI:** 10.1136/bmjgh-2015-000020

**Published:** 2016-07-07

**Authors:** David R Ellard, Wanangwa Chimwaza, David Davies, Doug Simkiss, Francis Kamwendo, Chisale Mhango, Siobhan Quenby, Ngianga-bakwin Kandala, Joseph Paul O'Hare

**Affiliations:** 1 Warwick Clinical Trials Unit, Division of Health Sciences, Warwick Medical School, The University of Warwick, Coventry, UK; 2 Malawi University, College of Medicine, Blantyre, Malawi; 3 Educational Development & Research Team, Warwick Medical School, The University of Warwick, Coventry, UK; 4 Division of Mental Health & Wellbeing, Warwick Medical School, The University of Warwick, Coventry, UK; 5 Obstetrics and Gynaecology Department, Malawi University, College of Medicine, Blantyre, Malawi; 6 College of Medicine, University of Malawi, Blantyre, Malawi; 7 Division of Reproductive Health, Warwick Medical School, The University of Warwick, Coventry, UK; 8 Faculty of Engineering and Environment, Department of Mathematics and Information sciences, Northumbria University, Newcastle upon Tyne, UK; 9 Division of Metabolic & Vascular Health, Warwick Medical School, The University of Warwick, Coventry, UK

## Abstract

**Background:**

The ETATMBA (Enhancing Training And Technology for Mothers and Babies in Africa) project-trained associate clinicians (ACs/clinical officers) as advanced clinical leaders in emergency obstetric and neonatal care. This trial aimed to evaluate the impact of training on obstetric health outcomes in Malawi.

**Method:**

A cluster randomised controlled trial with 14 districts of Malawi (8 intervention, 6 control) as units of randomisation. Intervention districts housed the 46 ACs who received the training programme. The primary outcome was district (health facility-based) perinatal mortality rates. Secondary outcomes included maternal mortality ratios, neonatal mortality rate, obstetric and birth variables. The study period was 2011–2013. Mortality rates/ratios were examined using an interrupted time series (ITS) to identify trends over time.

**Results:**

The ITS reveals an improving trend in perinatal mortality across both groups, but better in the control group (intervention, effect −3.58, SE 2.65, CI (−9.85 to 2.69), p=0.20; control, effect −17.79, SE 6.83, CI (−33.95 to −1.64), p=0.03). Maternal mortality ratios are seen to have improved in intervention districts while worsening in the control districts (intervention, effect −38.11, SE 50.30, CI (−157.06 to 80.84), p=0.47; control, effect 11.55, SE 87.72, CI (−195.87 to 218.98), p=0.90). There was a 31% drop in neonatal mortality rate in intervention districts while in control districts, the rate rises by 2%. There are no significant differences in the other secondary outcomes.

**Conclusions:**

This is one of the first randomised studies looking at the effect of structured training on health outcomes in this setting. Notwithstanding a number of limitations, this study suggests that up-skilling this cadre is possible, and could impact positively on health outcomes.

**Trial registration number:**

ISRCTN63294155; Results.

**Key questions**

**What is already known about this topic?**

- There is a global shortage of health professionals, particularly in sub-Saharan Africa; associate clinicians (ACs; clinical officers, in Malawi; previously non-physician clinicians) are in the frontline of healthcare in countries like Malawi.
- Countries, like Malawi, while making some advances, are struggling to achieve the United Nations Millennium Developmental Goals for child mortality and maternal health (MDG 4 and 5). In 2007, the Malawian Ministry of Health, recognising some of these issues, set out a National Road Map for accelerating the reduction of maternal and child mortality, and achieving MGD 5.
- Up-skilling ACs may provide a solution to some of these issues.

**What are the new findings?**

- This is one of the first trials taking an in-depth look at the impact on health outcomes in districts across central and northern Malawi with a programme of knowledge, skills and clinical leadership training for ACs. The trial's aim is to see if the up-skilling of this important cadre of health workers can impact on district maternal mortality rates, perinatal mortality rates, and key obstetric and birth complications. The results show some very positive trends.
- It is our belief that as the trainees share their new skills and knowledge, this positive impact will grow.

**Recommendations for policy**

- This cadre is an important component in helping to relieve the chronic shortages of trained medical professionals in sub-Saharan Africa, and for helping countries move towards realisation of the millennium development goals. Further evaluations of the up-skilling of this cadre are needed.

## Background

Many African countries, like Malawi, have a cadre of health workers called associate clinicians (ACs), previously non-physician clinicians, who are trained in basic medical diagnosis and treatment.^1^ ACs are often the most experienced health worker in hospitals across the country. Many of these ACs specialise in emergency obstetric and neonatal care (EmONC), and are in the frontline providing care for mothers and babies. The value of ACs cannot be understated; it will take many more years before countries like Malawi and most countries in sub-Saharan Africa have enough doctors. ACs, known as clinical officers in Malawi, are providing essential, valuable, safe and effective EmONC services across sub-Saharan African countries.^1–6^ Recent work has shown that while they are a valuable resource, they often feel undervalued and undersupported, and this has an impact on their performance and retention.^7^ ^8^ The WHO recognises the important role this cadre of health workers can play in maternal and newborn healthcare, and has made recommendations so that their role can be elevated.^9^ Major surveys consistently show that extra training and support can improve task shifting, and reduce maternal and neonatal mortality and morbidity in the areas where extra training and support have been piloted.^3^ ^4^ ^6^ Training skilled attendants to prevent, detect and manage major obstetric complications, including undertaking emergency caesarean surgery in complicated deliveries, is arguably the single most important factor in preventing maternal deaths and protecting the human rights of women.^2–4^ ^6^ To be effective, ACs need the appropriate knowledge, skills, equipment, drugs and the technology essential for managing obstetric complications in rural or deprived communities.

In 2011, the European Commission FP7 funded the enhancing of human resources and use of appropriate technologies for maternal and perinatal survival in sub-Saharan Africa (ETATMBA, Enhancing Training And Technology for Mothers and Babies in Africa) project. The project set out to implement and evaluate a programme of locally based clinical service improvement in Tanzania and Malawi. A cohort of EmONC ACs (in both countries) were provided with a programme of evidenced-based skills, knowledge and clinical leadership training delivered by European and African specialist clinicians and academicians. Trainees had links to specialist support and in Malawi, two UK-based obstetricians, at specialist registrar level with 5 years of clinical experience, worked alongside the ACs for 2 weeks in their districts, providing peer support and sharing of skills and knowledge.^10^

Here we report on the evaluation of the impact of the training in Malawi. The main aim is to evaluate the impact of the ETATMBA training on health outcomes (maternal and perinatal morbidity and mortality) by comparing districts where trainees were based with districts where there were no ETATMBA trainees.

## Methods

### Trial design

A cluster randomised controlled trial (RCT) with a 1:1 allocation. However, for the purpose of analyses, we adopted a quasiexperimental research design, as we were using district population-level data, in order to capture the longitudinal effects of the intervention through regression modelling. The main advantage of this approach is that it makes full use of the longitudinal nature of the data, and accounts for preintervention trends.^11^ ^12^

### Study setting

The study was conducted in districts within the central and northern regions of Malawi. There were a total of 14 districts (clusters) in these regions which were randomised to either intervention districts (where ETATMBA trainees were based) or control districts.

### Participants

There are no ‘participants’ per se in this study; the trainees who were recruited in the district are in reality the intervention group.

### Intervention

**Figure 1:**
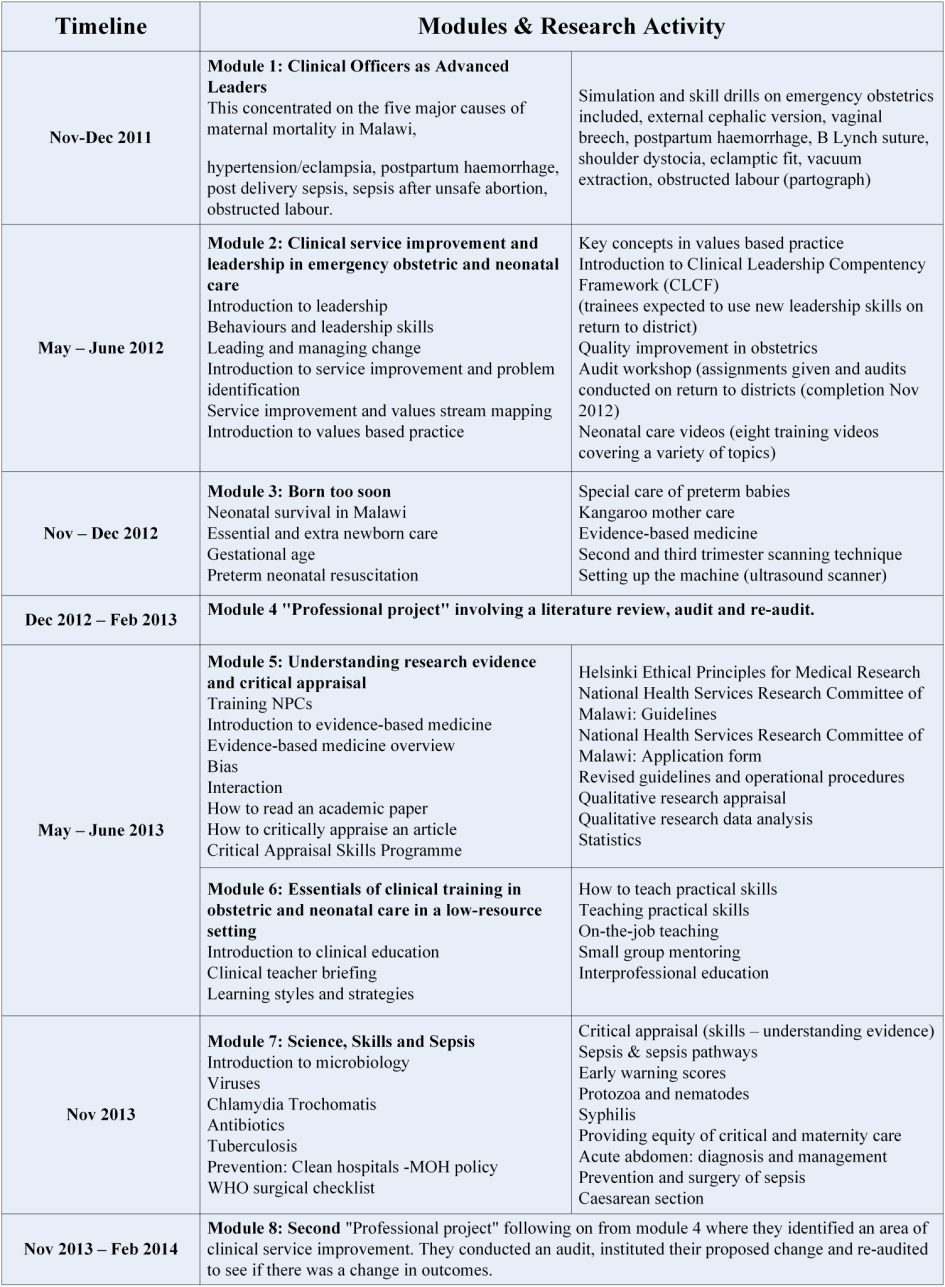
An overview of the ETATMBA training modules and when these were delivered. ETATMBA, Enhancing Training And Technology for Mothers and Babies in Africa; NPC, non-physician clinician.

Following randomisation, ACs within the intervention districts were invited to enrol on the ETATMBA training programme. The training was delivered between late 2011 and early 2014, and consisted of eight modules with mentoring and support offered between modules. The training was accredited at undergraduate degree level, and was specifically aimed at improving skills and knowledge related to EmONC and neonatal care, and had a strong clinical leadership component. Figure 1 outlines the training modules and their timeline. More detailed information on recruitment and modules is available elsewhere,^10^ ^13^ in the online supplementary appendix. Fifty-four trainees were included in the study, and they were based in the eight intervention districts. In these districts, a minimum of two ETATMBA trainees were included. The control districts received no ETATMBA trainees.

10.1136/bmjgh-2015-000020.supp1 supplementary appendix

### Outcomes

Facility-based perinatal mortality (PNM; pragmatically defined in this study as fresh stillbirths and neonatal deaths before discharge from the healthcare facility) is our primary outcome, and our secondary outcomes were facility-based maternal mortality, neonatal mortality, stillbirths, postpartum haemorrhage, caesarean section, eclampsia and sepsis. All of these outcomes were the numbers of events as recorded in maternity records by clinical staff within the health facilities. Each facility within a district records birth events in a maternity record book. This book is collated monthly and summarised, presenting a sum total of each of the variables from the book (eg, number of births, number of women with pre-eclampsia, number of stillbirths (macerated and fresh), etc) and sent to a central point within the district for reporting to the Ministry of Health. It is from these summary records that we gathered our data from each of the districts.

### Power calculation

In this study, we based our power calculation on a neonatal mortality rate of 30 per 1000 live births.^14^ The study was powered to detect a 20% difference between intervention and control districts in PNM rate (neonate survival until discharge from facility). The study has 0.80 power to detect the 20% difference with an α of 0.05. This was based on each of the trainees being exposed to 700 birth events in each of the eight intervention districts.

### Randomisation

Using data from a 2011 Republic of Malawi Ministry of Health report,^15^ the study statistician looked at each of the 14 districts in terms of maternal deaths, stillbirths and neonatal deaths per 1000 (population), ranking each district for each variable. Summing these three ranks gave a score for each of the 14 districts. The 14 districts were then placed in two strata based on a median split of the ranked score (of which there were 7 in each). The strata represent high and low ranked districts. Using a random number generator in STATA software, four districts were randomised to the intervention from each strata.

### Data analysis

Descriptive statistics were generated for all variables, and PNM rates (per 1000 live births) and maternal mortality ratios (per 100 000 live births) were calculated (MS Excel and SPSS V.22) based on the number of birth events.

The primary outcome, PNM rates and maternal mortality ratios were examined using an interrupted time series (ITS). The time series looked at quarterly periods across the 3 years with the intervention (interruption) being introduced in the first quarter of 2012 (giving a preinterruption slope of 12 months prior to any training/exposure). The ITS, in our case, is a robust technique having the ability to evaluate both intended and unintended consequences of interventions, such as significant challenges, not least the confounding influence of training programmes in control districts that might have affected the intervention. The ITS (statistical comparison of time trends preintervention and postintervention) was carried out using SPSS (V.22). Autoregressive integrated moving average models were generated for the primary variable and maternal mortality ratios for intervention and control districts. Effects are reported from the slope of the regression line preintervention and postintervention (overall), and at 3-month intervals for 21 months.^11^ ^12^ CIs (95%) are calculated for all effects. In addition, to aid the comparison between intervention and control districts, percentages of the absolute effects are calculated as ‘relative effects’, that is, 100×(actual effect)/(predicted effect)**−**(actual effect).

Data are presented as tables, figures or charts, as appropriate.

Data were transcribed from records held at the district hospitals. Data represented the quarterly figures for a particular district and in total, we collected data for three whole years (2011–2013 inclusive). Quarterly data from January 2011 to January 2012 (five quarters) represents the pre-ETATMBA training period. The remaining seven quarters up to December 2013 was the follow-up period. The outcomes chosen were linked to elements of the training provided to the ACs, and are data that are routinely recorded and stored.

### Deviations from original protocol

Owing to unforeseen circumstances, we needed to adjust our protocol slightly once we started the study. We have, within this paper, clarified our power calculation and randomisation as there was some confusion with the original protocol. While our pragmatic decision was to split Lilongwe into two because of its size, in reality this proved impossible; hence, we ended up with eight intervention districts (Lilongwe, Nkhotakota, Ntcheu, Chitipa, Karonga, Mzimba/Mzuzu, Kasungu and Rumphi) and six control districts (Dedza, Dowa, Mchinji, Ntchisi, Salima and Nkhata Bay).

We stated in our protocol that primary data would be extracted from the maternity logs (Malawi Ministry of Health Maternity Register, V.2 (July 2008)) at the district hospital and also the summary data for all other facilities within the district, which were also held there. Data were collected at three points in time from all districts.^16^ Local (project-related) and national (Government-related) resource issues early in the project period forced us to change a number of things. First, it was impractical within our resource constraints to extract data directly from the registers; therefore, we collected the monthly or quarterly summary data for the whole district from the district hospital. Second, our data collection was carried out in two rather than the three visits that was originally planned.

### Ethics

The study was reviewed and approved by the Biomedical Research Ethics Committee (BREC) at the University of Warwick, UK (143/09/2011), and the College of Medicine Research Ethics Committee (COMREC), Malawi (P.07/11/1102). It had also the approval and support of the Malawi Ministry of Health.

### Role of the funding source

The funders of this study had no input into the design and delivery of the programme and were not involved in any way with the studies data and its analysis.

## Results

Table 1 shows the total birth events for each district, and overall for the intervention and control districts. Here we see that the intervention districts had almost twice as many birth events when compared with the control over the study period (eg, in 2013, 155 425 compared with 79 437). There were missing data from one district and for reasons unknown, in 2011, Dowa appears to have 8000 more births (these data were checked and verified).

**Table 1:** Total birth events within health facilities over the study period 2011–2013 by district

Districts	Number of health facilities*	I/C†	Total births (n)		
			2011	2012	2013
Chitipa	9	I	7186	8308	8173
Karonga	15	I	7018	6088	8240
Kasungu	21	I	14 190	14 480	17 761
Mzimba	40	I	28 095	29 198	28 800
Ntcheu	22	I	18 732	17 290	18 245
Rumphi	15	I	8316	7732	8191
Nkhotakota	17	I	9940	9966	10 031
Lilongwe	54	I	53 810	55 922	55 984
Dedza	27	C	21 627	22 685	23 501
Dowa	20	C	20 417	12 647	12 986
Mchinji	13	C	19 373	20 486	17 512
Nkhata bay	17	C	6193	5893	6223
Ntchisi	10	C	5855	5986	6920
Salima	14	C	MD	11 148	12 295
Intervention (I)			147 287	148 984	155 425
Control (C)			73 465	78 845	79 437

*This is the number of health facilities that are included in the data for each of the districts.

†C, control districts; I, intervention districts.

MD, missing data.

Below we present the ITS analyses which explores the two primary mortality figures in more detail followed by the actual mortality figures, and the key obstetric and birth variables. A full data set, of all variables, broken down to individual districts is provided as an online supplementary appendix.

### Facility-based PNM (ITS)

**Figure 2:**
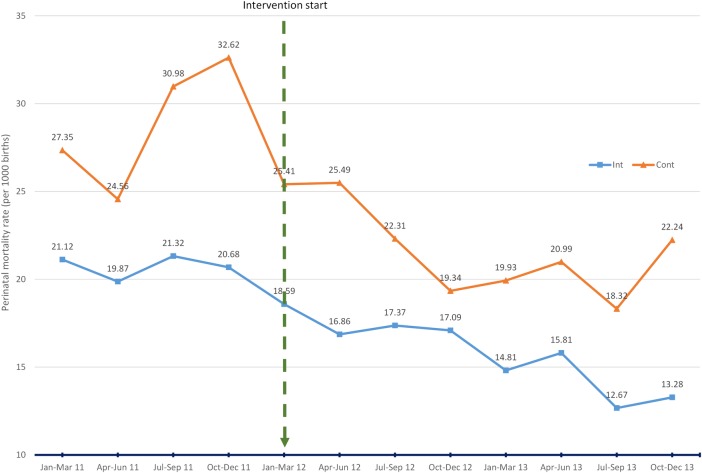
Interrupted time series: district health facility perinatal mortality rate (per 1000 births) comparing intervention districts (Int) with control districts (Cont).

Figure 2 and table 2 show the results for the ITS of PNM rates, our primary outcome. For the first quarter of 2011 (first data point) in the intervention and control districts, rates were 21.12 and 27.35 (per 1000 births), respectively. The rates reduce by 0.407 and 0.0966 points, respectively, per quarter prior to the intervention (at the end of the fourth quarter of 2011). When this trend is taken into account in the ITS analyses, it is uncertain what, if any, impact the ETATMBA training has had on the districts PNM rate. There is a consistent downward trend in the intervention and control districts throughout the follow-up period. However, the decline in the rate in the control district, which starts from a higher point than the intervention districts, is significantly better at p=0.05 at all points.

**Table 2:** Effects from the ITS models for district facility-based perinatal mortality rates, comparing intervention with control

	Intervention					Control				
	Effect	SE	CI 95%	p Value	Relative effect (%)	Effect	SE	CI 95%	p Value	Relative effect (%)
3 months	−1.36	1.18	(−4.16 to 1.43)	0.286	−7	−9.14	2.98	(−16.18 to −2.09)	0.018	−27
6 months	−1.73	1.36	(−4.94 to 148)	0.243	−9	−10.58	3.45	(−18.73 to −2.43)	0.018	−34
9 months	−2.10	1.58	(−5.83 to 1.63)	0.224	−12	−12.02	4.03	(−21.54 to −2.50)	0.020	−36
12 months	−2.47	1.83	(−6.79 to 1.84)	0.218	−14	−13.46	4.68	(−24.52 to −2.41)	0.024	−38
15 months	−2.84	2.09	(−7.78 to 2.10)	0.216	−16	−14.91	5.37	(−27.60 to −2.21)	0.027	−42
18 months	−3.21	2.37	(−8.81 to 2.38)	0.217	−19	−16.35	6.09	(−30.75 to −1.95)	0.031	−46
21 months	−3.58	2.65	(−9.85 to 2.69)	0.219	−21	−17.79	6.83	(−33.95 to −1.64)	0.035	−47

Effect—estimate of effect from ARIMA ITS model.

Relative effect, percentage change (compared with preslope trend).

ITS, interrupted time series.

Comparison of the standardised relative effects illustrates the greater decline in the control district rates compared with the intervention (eg, at 18 months, 46% decline in control compared with 19% in intervention; table 2). Figure 2 illustrates the PNM rates across the lifetime of the trial showing the downward trends with slight increases at the last data point (the last quarter of 2013), but rates at this time are clearly much lower in the intervention districts compared with the control districts (13.28 and 22.24, respectively).

### Facility-based maternal mortality (ITS)

**Figure 3:**
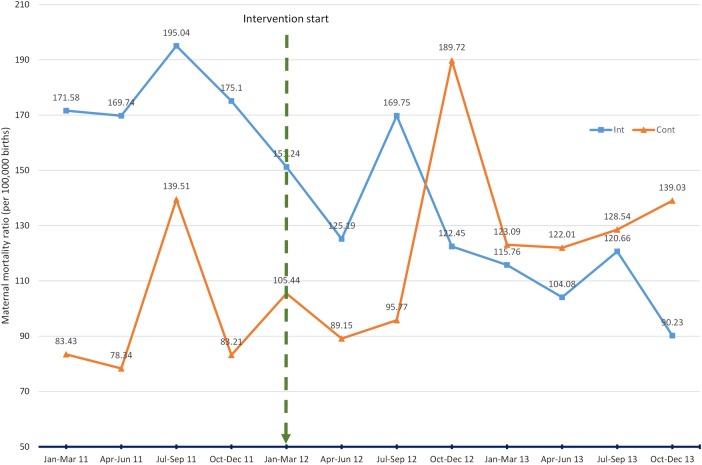
Interrupted time series: district health facility maternal mortality ratio (per 100 000 births), comparing intervention districts (Int) with control districts (Cont).

Figure 3 and table 3 show the results for the ITS of maternal mortality rates. Maternal mortality ratios for the first quarter of 2011 (first data point) in the intervention and control districts were 171.58 and 83.43 (per 100 000 live births), respectively. It is clear that the intervention districts are reporting a higher ratio than the control at the time of the intervention start, the first quarter of 2012, with ratios of 151.24 and 105.44, respectively. The ratios do reduce by 6.879 and 7.304 points, respectively, per quarter prior to the intervention (at the end of the fourth quarter of 2011). When we take this trend into account, there is a consistent reduction in maternal mortality ratio in the intervention districts while in the control districts there is a steady increase. The CIs around the effect estimates are wide and make us cautious in overinterpreting this result (table 3).

**Table 3:** Effects from the ITS models for district facility-based maternal mortality ratios, comparing intervention with control

	Intervention					Control				
	Effect	SE	CI 95%	p Value	Relative effect (%)	Effect	SE	CI 95%	p Value	Relative effect (%)
3 months	−14.87	22.78	(−68.73 to 38.99)	0.535	−9	4.85	36.88	(−82.37 to 92.06)	0.899	4
6 months	−18.68	26.03	(−80.24 to 42.88)	0.496	−12	5.97	42.49	(−94.50 to 106.43)	0.892	5
9 months	−22.49	30.13	(−93.74 to 48.77)	0.480	−16	7.08	49.60	(−110.19 to 124.36)	0.890	6
12 months	−26.30	34.78	(−108.54 to 55.94)	0.474	−18	8.20	57.66	(−128.13 to 144.53)	0.891	8
15 months	−30.11	39.79	(−124.19 to 63.96)	0.474	−21	9.32	66.32	(−147.51 to 166.14)	0.892	8
18 months	−33.93	45.03	(−140.40 to 72.54)	0.476	−24	10.44	75.39	(−167.82 to 188.70)	0.894	8
21 months	−38.11	50.30	(−157.06 to 80.84)	0.473	−29	11.55	87.72	(−195.87 to 218.98)	0.895	9

Effect—estimate of effect from ARIMA ITS model.

Relative effect, percentage change (compared with preslope trend).

ITS, interrupted time series.

However, comparison of the standardised relative effects shows that there is a consistent reduction in the intervention districts compared with a consistent increase in the control districts (table 3). Figure 3 illustrates the maternal mortality ratios across the lifetime of the trial and shows that starting from a higher baseline, the intervention districts maternal mortality ratio reduces. For the control districts, starting from a lower baseline, the maternal mortality ratio increases throughout the trial period. Indeed, the absolute ratio in the intervention and control districts by the final quarter of 2013 were 90.23, a reduction of 61.01 points from intervention start and 139.03, an increase of 33.6 points from intervention start.

Table 4 shows the actual mortality rates/ratios over the lifetime of the project. In PNM rates, there was an improvement of 32% in the intervention districts compared with only 30% in the control, with intervention districts having consistently lower PNM rates than control. The rate of early neonatal deaths, a key component of PNM, decreases over the 3 years of the project in the intervention districts (from 5.8 in 2011 to 4 in 2013, per 1000 births) by 31%; in the control districts, the rate is higher overall and has increased by 2% in 2013 when compared with 2011 (see table 4). Fresh stillbirth rates reduce by nearly 46% in the control districts and by only 32% in the intervention districts. Maternal mortality in the intervention districts reduces by 40%; however, in the control districts, the ratio increases by 31% by the end of the project.

**Table 4:** District facility-based mortality ratios/rates per year for 2011–2013

		Intervention districts N=8			Control districts N=6	
Variable and year	Births n=	Events n=	Rates*/ratio†	Births n=	Events n=	Rates*/ratio†
Perinatal mortality (PNM) and PNM rate per 1000 births						
2011‡	147 287	3057	20.8	73 465	2130	29.0
2012	148 984	2599	17.4	78 845	1825	23.2
2013	155 425	2840	14.2	79 437	1612	20.3
Early neonatal death (ND) and ND rate per 1000 births						
2011‡	147 287	859	5.8	73 465	703	9.6
2012	148 984	788	5.3	78 845	694	8.8
2013	155 425	625	4.0	79 437	778	9.8
Stillbirths fresh (SBF) and SBF rate per 1000 births						
2011‡	147 287	2198	14.9	73 465	1427	19.4
2012	148 984	1811	12.2	78 845	1131	14.3
2013	155 425	1574	10.1	79 437	834	10.5
Stillbirths macerated (SBM) and SBM rate per 1000 births						
2011‡	147 287	880	6	73 465	569	7.7
2012	148 984	856	5.7	78 845	641	8.1
2013	155 425	1063	6.2	79 437	633	8
Maternal mortality (MM) and MM ratio per 100.000 births						
2011‡	147 287	262	177.9	73 465	72	98.0
2012	148 984	211	141.6	78 845	94	119.2
2013	155 425	185	107.5	79 437	102	128.4

*Rates are calculated as number of events divided by total births, multiplied by 1000.

†Ratios for MM are calculated as number of events divided by total births, multiplied by 100 000.

‡Missing data from one control district for the whole of 2011.

### Obstetric complications and caesarean sections

Table 5 shows the number and rate of key obstetric complications over the trials lifetime. There are increases in the cases/rates of prolonged labour across both intervention and control districts. Cases of (pre)-eclampsia remain similar throughout as do many of the other complications.

**Table 5:** Comparison of key obstetric complications (facility based) by year

		Intervention districts N=8			Control districts N=6*	
Variable and year	Births n=	Events n=	Rate†	Births n=	Events n=	rate†
Prolonged labour (PL) and PL rate per 1000 births						
2011*	147 287	2864	19.5	73 465	2627	35.8
2012	148 984	2931	19.7	78 845	2690	34.2
2013	155 425	5098	32.8	79 437	3132	39.4
(Pre-)eclampsia (pE) and pE rate per 1000 births						
2011*	147 287	593	4.0	73 465	406	5.5
2012	148 984	947	6.4	78 845	462	5.9
2013	155 425	895	5.8	79 437	484	6.1
Sepsis (maternal) (Sm) and Sm rate per 1000 births						
2011*	147 287	153	1.0	73 465	91	1.2
2012	148 984	209	1.4	78 845	92	1.2
2013	155 425	198	1.27	79 437	69	0.9
Ruptured uterus (RU) and RU rate per 1000 births						
2011*	147 287	143	1.0	73 465	97	1.3
2012	148 984	127	0.9	78 845	97	1.2
2013	155 425	216	1.4	79 437	75	1.0
Haemorrhage (H) and H rate per 1000 births						
2011*	147 287	1705	11.6	73 465	1125	15.3
2012	148 984	2821	19.0	78 845	1518	19.3
2013	155 425	2291	15.8	79 437	1404	18.7

*Missing data from one control district for the whole of 2011.

†Rates are calculated as number of events divided by total births, multiplied by 1000.

### Birth complications

Table 6 shows the number and rate of key birth complications and caesarean sections over the lifetime of the trial. Of note, here is an increase in the reported rates of neonatal asphyxia, which increases in the intervention and control districts. Caesarean sections (cases/percentages) increase in the intervention district while remaining fairly constant in the control districts.

**Table 6:** Comparison of key birth complications and caesarean sections (facility based) by year

		Intervention districts N=8			Control districts N=6	
Variable and year	Births n=	n	Rate*	Births n=	n	Rate*
Premature birth (PB) and PB rate per 1000 births						
2011†	147 287	3025	20.6	73 465	2095	28.6
2012	148 984	2424	16.3	78 845	1996	25.3
2013	155 425	3036	19.5	79 437	2065	26
Low birthweight (LBW) and LBW rate per 1000 births‡						
2011†	147 287	2949	20.0	73 465	3723	50.7
2012	148 984	2968	20.0	78 845	3272	41.5
2013	155 425	4517	29.1	79 437	3042	38.3
Neonatal asphyxia (NA) and NA rate per 1000 births						
2011†	147 287	2301	15.6	73 465	1425	19.4
2012	148 984	2704	18.2	78 845	2010	25.5
2013	155 425	4104	26.4	79 437	2710	34.1
Neonatal sepsis (NS) and NS rate per 1000 births						
2011†	147 287	454	3.1	73 465	587	8.0
2012	148 984	707	4.8	78 845	415	5.3
2013	155 425	892	5.7	79 437	543	6.8
Vacuum extraction (VE) and VE rate per 1000 births						
2011†	147 287	2056	14.0	73 465	691	9.4
2012	148 984	2165	10.2	78 845	1052	13.3
2013	155 425	5601	13.9	79 437	1235	15.6
Breech delivery (BD) and BD rate per 1000 births						
2011†	147 287	2165	14.7	73 465	2166	29.5
2012	148 984	1603	10.8	78 845	1943	24.6
2013	155 425	2035	13.1	79 437	1618	20.4
Caesarean sections (CS) and CS percentage§						
		n	%§		n	%§
2011†	147 287	5601	3.8	73 465	3923	5.3
2012	148 984	6319	4.2	78 845	3942	5.0
2013	155 425	9368	6.0	79 437	4163	5.2

*Rates are calculated as number of events divided by total births, multiplied by 1000.

†Missing data from one control district for the whole of 2011.

‡Birthweight <2500 g.

§Percentage=number of caesarean section/number births×100.

### ETATMBA trainee outcomes

Fifty-four trainees were recruited, representing 67% (54/81) of the ACs working in emergency obstetric and neonatal care (EmONC) in the intervention districts. Of those recruited, 46 (85%) remained in the training programme till the end, 25 from the central region of Malawi drawn from nine hospitals (district and central hospitals) and 21 from the northern region drawn from six hospitals (district and central hospitals). One of the smaller districts in the northern region had one ETATMBA trainee working in its district hospital. Nearly all the trainees were male, with only two females. All of the 46 trainees completed the training and were awarded their degree in late 2014.

## Discussion

The main aims of this study were to evaluate the impact on health outcomes (perinatal and maternal morbidity and mortality) of the ETATMBA training programme. We are pleased to see an overall reduction in PNM rates (control and intervention). Attributing this reduction to our training is complex as reductions are statistically better in control districts. However, on closer examination of the early neonatal death rates (a key component of PNM), we find a fall by 31% (per 1000 births) in the intervention districts and a 2% increase in the control districts over the 3 years of the project. There were twice as many birth events within the intervention districts compared with control over the lifetime of the project implying that our trainees were exposed to more birth events. Our original assumption was that training would reduce perinatal deaths in the intervention district more than control through early intervention (eg, effective resuscitation) and enhanced care of the neonate. We are cautious in interpreting these results as there are confounding factors, but evidence from our qualitative studies in Malawi and Tanzania do support the notion that our training has been effective.^13^

A problem with interpreting these data is that when resuscitating babies born in very poor condition, some cases end in reclassifying fresh stillbirths as neonatal deaths. The Helping Babies Breath initiative was occurring at the same time as the ETATMBA training programme across Malawi, and this effect of moving some fresh stillbirths to neonatal deaths occurred in the intervention and control districts and explains the drop in fresh stillbirths without a drop in neonatal deaths in the control districts. Importantly, in the intervention districts, both stillbirths and neonatal deaths decreased suggesting that our training (which included resuscitation and early neonatal care) ended in more successful resuscitations in the intervention districts.

For maternal mortality, we see a consistent reduction in ratio over the 3 years in the intervention districts compared with a gradual increase within control districts. This does suggest that the training was starting to have an impact. Our qualitative work gave strong indications that at the local level (in the facilities where a trainee worked), maternal mortality had reduced. It takes effective teams to prevent maternal deaths, and the combination of knowledge, practical and leadership training was effective.^13^ We again are cautious in interpreting this result, but we do feel that there is evidence that women's lives were saved as a result of our training.^13^

Caesarean sections increased in the intervention districts while remaining at a similar level in control districts. There are many factors that can contribute to this, but our process evaluation has shown us that trainees were more confident to intervene and work with the local team to provide a better outcome for mothers.^13^ In high-income countries, the increasing caesarean section rates are associated with an increase in maternal morbidity.^17^ In contrast, in low-income countries like Malawi where there are low caesarean section rates (∼5%), increasing the rates is associated with an increase in maternal and perinatal survival.^17^ Furthermore, our training included improving decision-making for indications of caesarean section, improving skills to avoid caesarean section (vaginal breech and vacuum delivery) and reducing complications from caesarean section (improved surgical technique, transverse skin incisions, antibiotic prophylaxis, use of WHO checklist, better management of intraoperative haemorrhage, improved communications between clinical team). Thus, the training may be responsible for the increased rate of caesarean section with a decrease in neonatal and maternal mortality.

As expected, carrying out an RCT in a sub-Saharan African setting has a number of limitations. Two major initiatives from US aid organisations were active across Malawi at the time of our study: Helping Babies Breathe and Kangaroo Mother Care. The control districts could not ethically be deprived of these initiatives, and this may have contributed as a major confounder to our results.

This was not a traditional cluster RCT design. There are no actual participants; the clusters are whole districts and intervention districts had a numbers of ACs who received the ETATMBA training, which did not happen in the control districts. Along with this, our reliance on locally recorded data in the districts health facilities is also a limitation. Indeed, we are cautious about overinterpreting results. Our researchers visited the districts at least twice, collecting the data and transcribing it from data pooled at the district centres/hospitals. While they were able to check some data against register entries, to do this for all facilities within a district would have required a huge number of research staff. While we do have missing data (and report this), we are confident that the data collected are an accurate reflection of events in all of the districts included in the trial. Our design using district data was pragmatic, and a limitation is that it will not give the true picture that would be obtained from population mortality surveillance; however, as all the trainees were practising at the district hospitals, we believe it provides a comparable measure of change.

Our original pragmatic plan to split Lilongwe into two was thwarted in reality, another limitation of this study. This is the largest district in the study with the largest concentration of ETATMBA trainees (nine) whose influence was potentially district-wide. This has somewhat unbalanced the study with many more birth events in the intervention districts overall (see online supplementary appendix). It may be considered that our randomisation failed because of the imbalance but, in good faith, we based our randomisation on previously published data. We believe that we ensured that all necessary steps in randomisation were taken care of by using the appropriate statistical programme and randomisation technique, which cover the control of variability, levels of randomisation, size of intervention arms and power to detect causal effects, as well as the many problems that commonly lead to postintervention bias. We present an accurate reflection of the reality.

We report elsewhere the high value the trainees placed on the mentoring and support they received from the visiting obstetricians and the ETATMBA team.^13^ A key part, and indeed a unique part, of the ETATMBA training was the integration of clinical leadership with clinical skills and knowledge teaching. Trainees were actively encouraged to take leadership roles, and cascade their new skills and knowledge within their districts, which included travelling out to other facilities. Our qualitative work provides evidence that cascading took place with more effective team work and commitment to improve facilities.^13^ However, countrywide political and infrastructure problems (eg, fuel shortages and electricity outages) early in the project did place restrictions on the trainees’ ability to cascade their skills (eg, travelling to other facilities). Our hopes are that the ripples from the training are far reaching and ongoing; hence, our objective is to look at district-wide outcomes. Indeed, results from the Tanzanian arm of the ETATMBA show similar outcomes.^18^ ^19^

A recent review looking at obstetric and newborn care capacity building in rural sub-Saharan Africa concluded that the millennium development goals will not be met, but suggests that simple packaged emergency obstetric interventions could have an impact in the future.^20^ Given the critical shortages of qualified obstetricians in countries like Malawi, some have started training cadres like ACs in obstetric surgery, particularly in rural/remote areas, with the hope of alleviating the problem.^21–26^ In Malawi, the health facilities have chronic shortages of essential equipment and drugs.^25^ This coupled with the health provider crisis demonstrate the challenges faced in trying to make an impact on health outcomes.^24–26^ Training in obstetric emergencies that includes high fidelity simulations, leadership training and clinical teaching can improve obstetric and neonatal outcomes; features included in the ETATMBA training.^27–29^

Very few RCTs of training with the outcome measure of obstetric complications have been reported and there is an urgent need for these.^29^ ^30^ Reported improvements in obstetric complications have been demonstrated in ‘before and after’ studies.^27^ ^29^ In low-income countries, the ability to control other variables (eg, confounders) is challenging and in judging the outcomes, one must take into account both qualitative and quantitative data over longer time frames and in context.

In conclusion, it is heartening to see the reductions in PNM, maternal and neonatal mortality rates/ratios presented here. We are cautious in our interpretation of these results. Attributing the changes to the ETATMBA training is complex. Although there have been a large number of challenges, we have successfully trained 46 ACs with advanced skills and knowledge in obstetric, neonatal care and clinical leadership. This training has had an excellent retention rate, and was well received by the trainees and those around them in the districts (all were awarded a BSc in International Obstetrics by the University of Warwick in October 2014). We feel that these results, supported by the qualitative evidence, show the training has changed practice, and as a result may have contributed to the positive downward trends in maternal and neonatal mortality rates, and an increase in numbers and quality of lifesaving obstetric interventions such as caesarean sections. Providing this cadre with the leadership and practical skills and knowledge, based on best evidence and tailored to be delivered in a low-resource setting, could be a practical solution to the doctor shortages in African countries. Our hope is that the ETATMBA trainees will have an enduring influence that will impact positively on future practice in Malawi and Tanzania, and will help in realisation of MDGs 4 and 5.
