# Supplementary material for: Up-skilling associate clinicians in Malawi in emergency obstetric, neonatal care and clinical leadership: the ETATMBA cluster randomised controlled trial
Source: BMJ Glob Health. 2016 Jul 7;1(1):e000020. doi: 10.1136/bmjgh-2015-000020 (PMC5321302; doi:10.1136/bmjgh-2015-000020)
Supplement: supplementary appendix [file bmjgh-2015-000020supp_appendix.pdf]

Up-skilling associate clinicians in Malawi in emergency obstetric, neonatal care and clinical leadership: the ETATMBA cluster randomised controlled trial

Supplementary appendix

Table of Contents

Page		
**2**		Brief outline of the ETATMBA Training (the intervention)

The following tables are additional data from the ETATMBA trial. Whilst in the main paper only totals for the intervention and control districts are presented here the data are broken-down to the ‘district’ level.

Page	Table number	Title
3	S1	Total Births, maternal death number and ratio (per 100,000 births)
4	S2	Perinatal mortality number and rate (per 1000 births)
5	S3	Neonatal deaths and stillbirths
6	S4	Obstetric complications
7	S4a	Obstetric complications continued
8	S5	Caesarean sections
9	S6	Birth complications
10	S6a	Birth complications continued
11	S6b	Birth complications continued

Brief outline of the ETATMBA Training (the intervention)

The training package was a 30 month programme of knowledge and skills training including mentoring of practice. In addition, two obstetricians at UK specialist registrar level with 5 years of clinical experience worked alongside the ACs, providing peer support in their district hospitals and sharing of skills and knowledge. The intervention is the training of ACs in specific skills consisting of in-depth theoretical review and demonstration of the prevention and management of four major causes of death of mothers, notably haemorrhage, sepsis, hypertensive diseases of pregnancy and unsafe abortion and the three most common causes of neonatal death (prematurity, sepsis and asphyxia). Key components of the training included leadership, advanced surgical skills for the management of emergency obstetric complications and the prevention of pre-term labour/birth. The aim of the training was to empower the trainee ACs and enhance their leadership skills, allowing them to think and to maximise the use of their limited material resources to provide the best care possible for their patients and facilities. Included in the training there were key components of clinical service improvement and values based practice, which can be expected to yield returns outside the study period. All trainees were assigned a mentor/tutor with whom they received support and both formal and informal discussions throughout the training via email and telephone. The training was accredited by the University of Warwick and successful completion of the training would provide the trainee with a BSc in International Obstetrics. This degree level training for this particular cadre was essential to enhance their status and self-confidence within the Malawi health services.

The training in Malawi was modular with support and mentorship provided between and within modules. The modules were:

- Module 1 "Emergency obstetrics and neonatal care in resource-limited setting"
- Module 2 "Leadership and service improvement"
- Module 3 "Born too soon"
- Module 4 "Professional project"
- Module 7 "Science, skills and sepsis"
- Module 8 "Second Professional Project"

Much more information can be found at http://www2.warwick.ac.uk/fac/med/about/global/etatmba/

Table S1. Total Births, maternal death number and ratio (per 100,000 births)

Districts		Total Births (n)			Maternal Deaths (n)			Maternal Mortality ratio per 100.000 births		
		2011	2012	2013	2011	2012	2013	2011	2012	2013
Chitipa	I	7186	8308	8173	15	9	4	208.74	108.33	48.94
Karonga	I	7018	6088	8240	16	3	3	227.99	49.28	36.41
Kasungu	I	14190	14480	17761	40	24	20	281.89	165.75	112.61
Mzimba	I	28095	29198	28800	23	27	28	81.87	92.47	97.22
Ntcheu	I	18732	17290	18245	22	16	15	117.45	92.54	82.21
Rumphi	I	8316	7732	8191	51	57	31	613.28	737.20	378.46
Nkhotakota	I	9940	9966	10031	34	17	15	342.05	170.58	149.54
Lilongwe	I	53810	55922	55984	61	58	51	113.36	103.72	91.10
Dedza	C	21627	22685	23501	11	8	15	50.86	35.27	63.83
Dowa	C	20417	12647	12986	11	12	8	53.88	94.88	61.60
Mchinji	C	19373	20486	17512	23	26	24	118.72	126.92	137.05
Nkhata bay	C	6193	5893	6223	14	11	15	226.06	186.66	241.04
Ntchisi	C	5855	5986	6920	13	25	28	222.03	417.64	404.62
Salima	C	MD	11148	12295	MD	12	12	MD	107.64	97.60
Intervention		147287	148984	155425	262	211	167	177.88	141.63	107.45
Control		73465	78845	79437	72	94	102	98.01	119.22	128.40

I = Intervention, C = Control. MD = Missing data

Table S2. Perinatal mortality number and rate (per 1000 births)

Districts		Total Births (n)			Perinatal Mortality (n)			Perinatal Mortality per 1000 births		
		2011	2012	2013	2011	2012	2013	2011	2012	2013
Chitipa	I	7186	8308	8173	100	89	40	13.92	10.71	4.89
Karonga	I	7018	6088	8240	132	147	127	18.81	24.16	15.41
Kasungu	I	14190	14480	17761	324	346	220	22.83	23.90	12.39
Mzimba	I	28095	29198	28800	211	268	216	7.51	9.18	7.50
Ntcheu	I	18732	17290	18245	177	143	147	9.45	8.27	8.06
Rumphi	I	8316	7732	8191	107	110	59	12.87	14.23	7.20
Nkhotakota	I	9940	9966	10031	256	233	151	25.75	23.38	15.05
Lilongwe	I	53810	55922	55984	1750	1263	1239	32.51	22.59	22.13
Dedza	C	21627	22685	23501	326	338	430	15.07	14.90	18.30
Dowa	C	20417	12647	12986	387	254	231	18.95	20.08	17.79
Mchinji	C	19373	20486	17512	1142	849	444	58.95	41.44	25.35
Nkhata bay	C	6193	5893	6223	200	168	193	32.29	28.51	31.01
Ntchisi	C	5855	5986	6920	75	64	139	12.81	10.69	20.09
Salima	C	MD	11148	12295	MD	152	175	MD	13.63	14.23
Intervention		147287	148984	155425	3057	2599	2199	20.76	17.44	14.15
Control		73465	78845	79437	2130	1825	1612	28.99	23.15	20.29

I = Intervention, C = Control. MD = Missing data

Table S3. Neonatal deaths and stillbirths

		Early neonatal death (ND)						Stillbirth fresh (SBF)						Stillbirth macerated (SBM)					
		n			ND rate per 1000 births			n			SBF rate per 1000 births			n			SBM rate per 1000 births		
	I/C	2011	2012	2013	2011	2012	2013	2011	2012	2013	2011	2012	2013	2011	2012	2013	2011	2012	2013
Chitipa	I	52	38	18	7.2	4.6	11.6	48	51	75	6.7	6.1	9.2	42	39	47	5.8	4.7	5.8
Karonga	I	71	88	86	10.	14.5	21.7	61	59	94	8.7	9.7	12.4	65	31	172	9.3	5.1	22.8
Kasungu	I	132	162	105	9.3	11.2	13.4	192	184	233	13.5	12.7	13.1	112	148	125	7.9	10.2	7.0
Mzimba	I	82	78	70	2.9	2.7	2.8	129	190	196	4.6	6.5	6.8	126	147	211	4.5	5.0	7.3
Ntcheu	I	21	14	12	1.1	0.8	0.7	156	129	135	8.3	7.5	7.4	87	81	102	4.6	4.7	5.6
Rumphi	I	59	59	27	7.1	7.6	3.3	48	51	32	5.8	6.6	3.9	34	62	28	4.1	8.0	3.4
Nkhotakota	I	112	91	54	11.3	9.1	8.7	144	142	133	14.5	14.2	13.3	36	52	72	3.6	5.2	7.2
Lilongwe	I	330	258	253	6.1	4.6	4.5	1420	1005	986	26.4	18.0	17.6	378	296	306	7.0	5.3	5.5
Dedza	C	128	148	185	5.9	6.5	7.9	198	190	245	9.2	8.4	10.4	164	149	155	7.6	6.6	6.6
Dowa	C	146	109	11	7.2	8.6	8.5	241	145	121	11.8	11.5	9.3	148	115	116	7.2	9.1	8.9
Mchinji	C	261	253	228	13.5	12.3	13.0	881	596	216	45.5	29.1	12.3	154	161	135	7.9	7.9	7.7
Nkhata bay	C	133	109	136	21.5	18.5	21.9	67	59	57	10.8	10.0	9.2	58	105	74	9.4	17.8	11.9
Ntchisi	C	35	18	49	6.0	3.0	7.1	40	46	90	6.8	7.7	13.0	45	57	62	7.7	9.5	9.0
Salima	C	MD	57	70	MD	5.1	5.7	MD	95	105	MD	8.5	8.5	MD	54	91	MD	4.8	7.4
Intervention		859	788	625	5.8	5.3	6.2	2198	1811	1884	14.9	12.2	12.2	880	856	1063	6.0	5.7	6.9
Control		703	694	778	9.6	8.8	9.8	1427	1131	834	19.4	14.3	10.5	569	641	633	7.7	8.1	8.0

I = Intervention, C = Control. MD = Missing data

Table S4. Obstetric complications

		Prolonged labor						(Pre-) Eclampsia						Sepsis (maternal)					
		n			Per 1000 births			n			Per 1000 births			n			Per 1000 births		
	I/C	2011	2012	2013	2011	2012	2013	2011	2012	2013	2011	2012	2013	2011	2012	2013	2011	2012	2013
Chitipa	I	110	80	222	15.31	9.63	27.16	11	9	33	1.53	1.08	4.04	12	4	5	1.67	0.48	0.61
Karonga	I	176	151	190	25.08	24.80	23.06	70	37	58	9.97	6.08	7.04	21	45	42	2.99	7.39	5.10
Kasungu	I	175	273	717	12.33	18.85	40.37	32	67	92	2.26	4.63	5.18	41	44	44	2.89	3.04	2.48
Mzimba	I	119	777	1239	4.24	26.61	43.02	68	189	241	2.42	6.47	8.37	32	34	32	1.14	1.16	1.11
Ntcheu	I	247	63	28	13.19	3.64	1.53	32	10	2	1.71	0.58	0.11	3	1	0	0.16	0.06	0.00
Rumphi	I	131	173	122	15.75	22.37	14.89	40	71	19	4.81	9.18	2.32	13	19	7	1.56	2.46	0.85
Nkhotakota	I	180	224	516	18.11	22.48	51.44	14	24	48	1.41	2.41	4.79	9	32	28	0.91	3.21	2.79
Lilongwe	I	1726	1190	2064	32.08	21.28	36.87	326	540	402	6.06	9.66	7.18	22	30	40	0.41	0.54	0.71
Dedza	C	653	797	948	30.19	35.13	40.34	89	98	151	4.12	4.32	6.43	5	17	14	0.23	0.75	0.60
Dowa	C	776	655	599	38.01	51.79	46.13	142	100	94	6.95	7.91	7.24	14	6	4	0.69	0.47	0.31
Mchinji	C	683	677	661	35.26	33.05	37.75	129	169	119	6.66	8.25	6.80	17	34	17	0.88	1.66	0.97
Nkhata bay	C	429	233	360	69.27	39.54	57.85	25	22	24	4.04	3.73	3.86	16	8	8	2.58	1.36	1.29
Ntchisi	C	86	127	286	14.69	21.22	41.33	21	35	23	3.59	5.85	3.32	39	24	5	6.66	4.01	0.72
Salima	C	MD	201	278	MD	18.03	22.61	MD	38	73	MD	3.41	5.94	MD	3	21	MD	0.27	1.71
Intervention		2864	2931	5098	19.45	19.67	32.80	593	947	895	4.03	6.36	5.76	153	209	198	1.04	1.40	1.27
Control		2627	2690	3132	35.76	34.12	39.43	406	462	484	5.53	5.86	6.09	91	92	69	1.24	1.17	0.87

I = Intervention, C = Control. MD = Missing data

Table S4a. Obstetric complications continued

		Ruptured uterus						Haemorrhage					
		n			Per 1000 births			n			Per 1000 births		
	I/C	2011	2012	2013	2011	2012	2013	2011	2012	2013	2011	2012	2013
Chitipa	I	7	2	12	0.97	0.24	1.47	21	10	111	2.92	5.90	13.58
Karonga	I	9	3	10	1.28	0.49	1.21	220	22	214	31.35	30.22	25.97
Kasungu	I	21	19	41	1.48	1.31	2.31	203	36	420	14.31	13.12	23.65
Mzimba	I	10	29	52	0.36	0.99	1.81	120	63	463	4.27	13.97	16.08
Ntcheu	I	7	2	0	0.37	0.12	0.00	135	22	22	7.21	1.27	1.21
Rumphi	I	11	18	3	1.32	2.33	0.37	84	55	125	10.10	16.16	15.26
Nkhotakota	I	20	8	28	2.01	0.80	2.79	78	108	108	7.85	10.84	10.77
Lilongwe	I	58	46	70	1.08	0.82	1.25	844	1735	828	15.68	31.03	14.79
Dedza	C	12	17	21	0.55	0.75	0.89	293	407	393	13.55	17.94	16.72
Dowa	C	31	25	13	1.52	1.98	1.00	294	200	209	14.40	15.81	16.09
Mchinji	C	40	33	13	2.06	1.61	0.74	324	421	456	16.72	20.55	26.04
Nkhata bay	C	9	4	4	1.45	0.68	0.64	122	232	MD	19.70	39.37	MD
Ntchisi	C	5	4	3	0.85	0.67	0.43	92	88	125	15.71	14.70	18.06
Salima	C	MD	14	21	MD	1.26	1.71	MD	170	221	MD	15.25	17.97
Intervention		143	127	216	0.97	0.85	1.39	1705	2821	2291	11.58	18.93	14.74
Control		97	97	75	1.32	1.23	0.94	1125	1518	1404	15.31	19.25	17.67

I = Intervention, C = Control.. MD = Missing data

Table S5 Caesarean sections

		Caesarean section					
		n			Percentage		
	I/C	2011	2012	2013	2011	2012	2013
Chitipa	I	357	141	445	5.0	1.7	5.4
Karonga	I	323	242	283	4.6	4.0	3.4
Kasungu	I	691	729	1059	4.9	5.0	6.0
Mzimba	I	773	1868	2496	2.8	6.4	8.7
Ntcheu	I	336	175	106	1.8	1.0	0.6
Rumphi	I	232	480	91	2.8	6.2	1.1
Nkhotakota	I	359	340	868	3.6	3.4	8.7
Lilongwe	I	2530	2344	4020	4.7	4.2	7.2
Dedza	C	982	774	933	4.5	3.4	4.0
Dowa	C	1335	1090	768	6.5	8.6	5.9
Mchinji	C	832	900	762	4.3	4.4	4.4
Nkhata bay	C	446	363	488	7.2	6.2	7.8
Ntchisi	C	328	443	487	5.6	7.4	7.0
Salima	C	MD	372	725	MD	3.3	5.9
Intervention		5601	6319	9368	3.8	4.2	6.0
Control		3923	3942	4163	5.3	5.0	5.2

Percentage = number CS/number births X 100
I = Intervention, C = Control. MD = Missing data

Table S6. Birth complications

		Vacuum extraction						Breech delivery					
		n			Per 1000 births			n			Per 1000 births		
	I/C	2011	2012	2013	2011	2012	2013	2011	2012	2013	2011	2012	2013
Chitipa	I	17	72	73	2.37	8.67	8.93	36	75	115	5.01	9.03	14.07
Karonga	I	22	83	76	3.13	13.63	9.22	164	101	128	23.37	16.59	15.53
Kasungu	I	338	212	304	23.82	14.64	17.12	355	261	304	25.02	18.02	17.12
Mzimba	I	106	157	311	3.77	5.38	10.80	161	332	431	5.73	11.37	14.97
Ntcheu	I	66	49	18	3.52	2.83	0.99	201	39	32	10.73	2.26	1.75
Rumphi	I	62	92	15	7.46	11.90	1.83	87	97	61	10.46	12.55	7.45
Nkhotakota	I	61	68	152	6.14	6.82	15.15	87	92	148	8.75	9.23	14.75
Lilongwe	I	1384	792	1216	25.72	14.16	21.72	1074	606	816	19.96	10.84	14.58
Dedza	C	201	193	291	9.29	8.51	12.38	483	413	479	22.33	18.21	20.38
Dowa	C	138	122	119	6.76	9.65	9.16	448	284	298	21.94	22.46	22.95
Mchinji	C	199	278	555	10.27	13.57	31.69	1043	869	376	53.84	42.42	21.47
Nkhata bay	C	136	114	140	21.96	19.34	22.50	93	97	96	15.02	16.46	15.43
Ntchisi	C	17	36	50	2.90	6.01	7.23	99	114	169	16.91	19.04	24.42
Salima	C	MD	309	80	MD	27.72	6.51	MD	166	200	MD	14.89	16.27
Intervention		2056	2165	5601	13.96	10.24	13.93	2165	1603	2035	14.70	10.76	13.09
Control		691	1052	1235	9.41	13.34	15.55	2166	1943	1618	29.48	24.64	20.37

I = Intervention, C = Control. MD = Missing data

Table S6a Birth complications continued

		Premature						Birth weight less than 2500g					
		n			Per 1000 births			n			Per 1000 births		
	I/C	2011	2012	2013	2011	2012	2013	2011	2012	2013	2011	2012	2013
Chitipa	I	46	87	182	6.40	10.47	22.27	33	192	232	4.59	23.11	28.39
Karonga	I	204	164	175	29.07	26.94	21.24	178	198	255	25.36	32.52	30.95
Kasungu	I	378	378	390	26.64	26.10	21.96	346	277	543	24.38	19.13	30.57
Mzimba	I	299	629	759	10.64	21.54	26.35	281	667	842	10.00	22.84	29.24
Ntcheu	I	339	40	36	18.10	2.31	1.97	250	115	64	13.35	6.65	3.51
Rumphi	I	119	152	72	14.31	19.66	8.79	111	157	63	13.35	20.31	7.69
Nkhotakota	I	140	140	212	14.08	14.05	21.13	132	140	456	13.28	14.05	45.46
Lilongwe	I	1500	834	1210	27.88	14.91	21.61	1618	1222	2062	30.07	21.85	36.83
Dedza	C	661	550	553	30.56	24.25	23.53	990	845	864	45.78	37.25	36.76
Dowa	C	584	384	343	28.60	30.36	26.41	761	589	592	37.27	46.57	45.59
Mchinji	C	570	623	577	29.42	30.41	32.95	1412	1059	522	72.88	51.69	29.81
Nkhata bay	C	167	129	152	26.97	21.89	24.43	280	244	388	45.21	41.41	62.35
Ntchisi	C	113	90	181	19.30	15.04	26.16	280	286	266	47.82	47.78	38.44
Salima	C	MD	220	259	MD	19.73	21.07	MD	249	410	MD	22.34	33.35
Intervention		3025	2424	3036	20.54	16.27	19.53	2949	2968	4517	20.02	19.92	29.06
Control		2095	1996	2065	28.52	25.32	26.00	3723	3272	3042	50.68	41.50	38.29

I = Intervention, C = Control. MD = Missing data

Table S6b Birth complications continued

		Asphyxia						Neonatal sepsis					
		n			Per 1000 births			n			Per 1000 births		
	I/C	2011	2012	2013	2011	2012	2013	2011	2012	2013	2011	2012	2013
Chitipa	I	30	97	244	4.17	11.68	29.85	1	46	63	0.14	5.54	7.71
Karonga	I	110	137	158	15.67	22.50	19.17	129	161	164	18.38	26.45	19.90
Kasungu	I	292	592	583	20.58	40.88	32.82	80	86	333	5.64	5.94	18.75
Mzimba	I	222	570	1025	7.90	19.52	35.59	68	158	149	2.42	5.41	5.17
Ntcheu	I	191	41	30	10.20	2.37	1.64	27	1	1	1.44	0.06	0.05
Rumphi	I	106	91	68	12.75	11.77	8.30	25	21	12	3.01	2.72	1.47
Nkhotakota	I	92	136	352	9.26	13.65	35.09	16	140	84	1.61	14.05	8.37
Lilongwe	I	1258	1040	1644	23.38	18.60	29.37	108	94	86	2.01	1.68	1.54
Dedza	C	343	537	830	15.86	23.67	35.32	26	65	47	1.20	2.87	2.00
Dowa	C	489	355	402	23.95	28.07	30.96	140	35	39	6.86	2.77	3.00
Mchinji	C	319	524	611	16.47	25.58	34.89	198	149	116	10.22	7.27	6.62
Nkhata bay	C	209	212	276	33.75	35.97	44.35	154	127	224	24.87	21.55	36.00
Ntchisi	C	65	129	284	11.10	21.55	41.04	69	12	7	11.78	2.00	1.01
Salima	C	MD	253	307	MD	22.69	24.97	MD	27	110	MD	2.42	8.95
Intervention		2301	2704	4104	15.62	18.15	26.41	454	707	892	3.08	4.75	5.74
Control		1425	2010	2710	19.40	25.49	34.12	587	415	543	7.99	5.26	6.84

I = Intervention, C = Control. MD = Missing data
